# Earlier and more uniform spring green-up linked to lower insect richness and biomass in temperate forests

**DOI:** 10.1038/s42003-023-05422-9

**Published:** 2023-11-07

**Authors:** Lars Uphus, Johannes Uhler, Cynthia Tobisch, Sandra Rojas-Botero, Marvin Lüpke, Caryl Benjamin, Jana Englmeier, Ute Fricke, Cristina Ganuza, Maria Haensel, Sarah Redlich, Jie Zhang, Jörg Müller, Annette Menzel

**Affiliations:** 1 Ecoclimatology, TUM School of Life Sciences, Technical University of Munich, Freising, Germany; 2 Field Station Fabrikschleichach, Department of Animal Ecology and Tropical Biology, Julius-Maximilians-Universität Würzburg, Rauhenebrach, Germany; 3 Restoration Ecology, TUM School of Life Sciences, Technical University of Munich, Freising, Germany; 4 Institute of Ecology and Landscape, Weihenstephan-Triesdorf University of Applied Sciences, Freising, Germany; 5 Department of Animal Ecology and Tropical Biology, Julius-Maximilians-Universität Würzburg, Würzburg, Germany; 6 Professorship of Ecological Services, Bayreuth Center of Ecology and Environmental Research (BayCEER), University of Bayreuth, Bayreuth, Germany; 7 Bavarian Forest National Park, Grafenau, Germany; 8 Institute for Advanced Study, Technical University of Munich, Garching, Germany

**Keywords:** Phenology, Food webs

## Abstract

Urbanization and agricultural intensification are considered the main causes of recent insect decline in temperate Europe, while direct climate warming effects are still ambiguous. Nonetheless, higher temperatures advance spring leaf emergence, which in turn may directly or indirectly affect insects. We therefore investigated how Sentinel-2-derived start of season (SOS) and its spatial variability (SV-SOS) are affected by spring temperature and whether these green-up variables can explain insect biomass and richness across a climate and land-use gradient in southern Germany. We found that the effects of both spring green-up variables on insect biomass and richness differed between land-use types, but were strongest in forests. Here, insect richness and biomass were higher with later green-up (SOS) and higher SV-SOS. In turn, higher spring temperatures advanced SOS, while SV-SOS was lower at warmer sites. We conclude that with a warming climate, insect biomass and richness in forests may be affected negatively due to earlier and more uniform green-up. Promising adaptation strategies should therefore focus on spatial variability in green-up in forests, thus plant species and structural diversity.

## Abstract (web summary)

Earlier spring green-up and its reduced spatial variability is negatively correlated to insect richness and biomass in temperate forests. Through this pathway, climate warming may indirectly contribute to a decline in insect richness and biomass.

## Introduction

In just 27 years (1989-2016), a 76% decline in insect biomass has been recorded in temperate Europe^1^. As insects fulfil many key functions (e.g. pollination, pest suppression, food supply for higher trophic levels) on which entire ecosystems and human activities are highly dependent^2^, it is urgent to understand the reasons for this marked decline. Until recently, urbanization and agricultural intensification were considered the main drivers^3–6^. However, according to Müller et al.^7^ changes in temperature and precipitation can also explain the decline, although the exact mechanisms are complex. During the flight period, temperatures were found to have neutral to positive effects on insect biomass^3,7,8^ and richness^3,8^, although extreme heat is found to be negative^9,10^. Winter temperatures, on the other hand, are found to negatively affect insect biomass^7^. One reason for this could be that, especially in life stages previous to the flight period, climate may impact insects differently by more indirect pathways^11^. In particular, prominent climate change-induced shifts in phenological events^12,13^, such as leaf emergence, flowering or insect egg hatching, may also affect insect fitness and diversity^9^.

Phenology is an important trait for many organisms, as the presence of consumers and their resources must match spatially and temporally to optimize fitness^14–16^. Since climate warming-related shifts differ among trophic levels^17–19^, trophic mismatches are expected to occur more frequently^15^. So far, however, mismatch effects on insect populations have only been studied for single species pairs, e.g. between spring ephemeral *Corydalis ambigua* and bumble bees, its pollinators^20^, and between oak and winter moth^21^, although this could be one of the main reasons for their decline^9,22^. With spring green-up, vegetative and floral resources become available for primary consumers, but their optimal quality is important as well^23^. Therefore, too early or too late green-up in relation to the presence of consumers can lead to trophic mismatch^24^. If, for example, phytophagous insect larvae hatch before leaf emergence, larvae will starve^24^. On the other hand, if the leaves emerge well before the larvae hatch, their food quality may be less optimal, as the freshly emerged leaves are the most digestible, richest in protein and, regarding woody species, contain the fewest protective chemicals^23,25^. In the widely studied *Operophtera brumata* (winter moth), late hatching relative to the green-up of its host plant affects population fitness more negatively than early hatching relative to green-up^26^. However, it is unknown whether this also applies for the total insect community, including species with other annual life cycles^27^ and higher trophic levels.

For both types of mismatch, the inherent spatial variability of spring green-up, e.g. by topographical, land-use, structural or species diversity, in the insect foraging horizon could act as a buffer by providing timely high-quality forage to primary consumers^28^. Although this has been suggested several times in the literature^14,29,30^, it has been empirically demonstrated only for larger herbivores such as caribou^31^ and red deer^32^. Surprisingly, there are still no studies directly linking phenological variability to insect demography (but see the studies of Oliff-Yang et al.^28^ and Hindle et al.^33^ for indirect effects on single species).

Thus, we assume that for the total insect community a higher spatial variability in green-up is beneficial and that the mean green-up date is of influence, but the direction of influence unsure because of two contradicting mechanisms. Furthermore, since phenological sensitivity generally decreases with increasing tropic levels^17–19^, we expect that for insects belonging to functional groups of higher trophic levels, e.g. predators, these effects are less pronounced than for insects of lower trophic levels, e.g. phytophagous insects and pollinators. However, for parasitoids, although also higher trophic levels, we expect pronounced effects similar to primary consumers, as they are often highly specialized and use host-plant cues^34^.

By using a space-for-time approach across ~180 sites in southern Germany^22,35^ and high-resolution remote sensing^36^, we were able to circumvent the previously limiting logistical and temporal constraints linked to insect sampling and phenological observations. The mean start of the season (mean SOS) and spatial variability of SOS (SV-SOS) during 2017-2019 were derived from 10 m Sentinel-2 pixels^37^ for 100 m radii around our study plots in Bavaria. These 179 plots were stratified over a mean annual temperature gradient from 5.0 to 10.3 °C and included different local land-use types (forests – meadows – arable fields – settlements) embedded within three regional land-use types (seminatural – agricultural – urban landscapes)^3,38^. In spring/summer 2019, 1293 bi-weekly samples of insect BIN richness (further ‘insect richness’ as a proxy for species richness), and biomass were retrieved from 179 malaise traps^3^. We added the green-up variables (mean SOS and SV-SOS), as well as plant species richness, to the generalized additive models from Uhler et al.^3^, in which the insect richness and biomass data were explained by land use and climate only, and further explored green-up effects between land-use classes and functional and taxonomic insect groups.

## Results

**Fig. 1:**
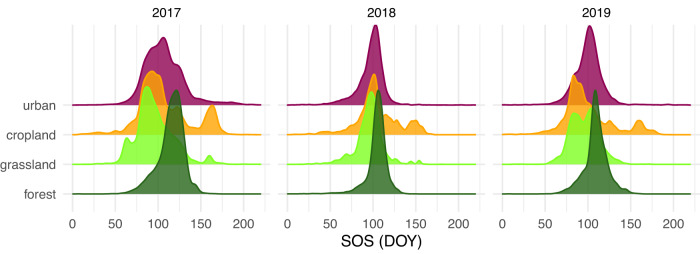
SOS distribution per year and land use type. Annual density ridges of SOS (DOY) in 2017-2019 for the four most dominant land use types (covering 99.99% of nonwater surface) in 100 m radii around the 179 study plots with Malaise traps on a climate and land-use gradient in Bavaria. “Semi-natural areas” (covering 0.01% of non-water surface) are not shown.

In 2017-2019, the mean SOS date (DOY) was 109.5 in forested areas (sd 14.7 d), 104.2 in cropland (sd 28.6 d), 96.0 (sd 18.7 d) in grassland, 101.7 (sd 20.2 d) in urban areas, i.e. with considerable differences between these land-use classes. Moreover, mean SOS dates also varied between years: mean DOY 107.8 (sd 24.0 days) in 2017, mean DOY 101.6 (sd 18.6 d) in 2018 and mean DOY 102.8 (sd 20.7 d) in 2019 (Fig. 1). How SOS differed among those land-use classes again also depended on the year and their March and April temperatures. For example, the mean air temperature on the plots in March 2017 (6.8 °C) was 2.7 °C warmer than in the other 2 years followed by an April (7.2 °C), which was 3.9 °C cooler, resulting in a rather high SOS difference between forest (later) and the other land-use classes (earlier). On the other hand, in 2018, with a relatively cold March (2.3 °C, ˗4.2 °C compared to other two years) and a warm April (12.6 °C, +4.3 °C compared to other two years), the SOS differences between land-use classes were relatively small (Fig. 1). In general, forest plots had the lowest SV-SOS (mean 9.7 d, range 2.4 to 20.4 d), indicating lower spatial variation in SOS dates. In contrast, arable field plots had the highest SV-SOS (mean 19.4 d, range 7.7 to 34.5 d).

**Fig. 2:**
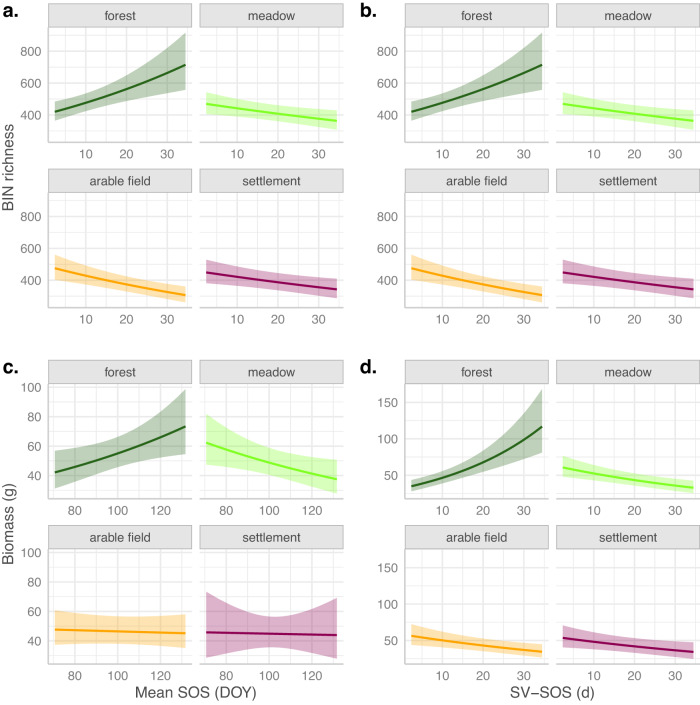
Partial effects of the green-up variables on insect BIN richness and Biomass. Effects were derived from generalized additive models (gams) on BIN richness (**a**, **b**) and on Biomass (**c**, **d**), in which we included regional land use and interactions of local land use with all green-up variables, with all climate variables and with species richness as fixed linear effects (see Table 1). ‘Day’ was used as smoothed effect and space as random effect. An offset of log(sampling days) was used to control for sampling period differences. For biomass, family = gaussian(link = “log”) and for richness, family = negative binomial were used. Insect data were recorded over a whole season (8 samplings) at 179 traps, resulting in 1214 observations for BIN Richness and 1293 observations for Biomass. **a**, **c**, partial effects of mean SOS. **b**, **d**, partial effects of SV-SOS.

In our generalized additive models (gams) to explain insect BIN richness and biomass by regional land use and interactions between local land use and green-up variables, climate variables, and plant species richness as fixed linear effects, we found strong distinctions between the forest plots and the other local land-use types. For forest plots, partial effects of both green-up variables were significant, where insect biomass and richness were increased with later mean SOS and higher SV-SOS (Table 1; Fig. 2). For arable field and meadow plots, insect richness was increased with later mean SOS as well. In contrast, biomass in meadow plots was decreased with later mean SOS. Biomass in arable field and settlement plots, as well as richness in settlement plots did not respond to mean SOS. For meadow, arable field and settlement plots, both richness and biomass were decreased with higher SV-SOS (Table 1; Fig. 2). Insect richness and biomass were both increased with higher local temperatures in all local land-use types, whereas with local humidity, richness was increased in arable field plots and decreased in settlement plots. With mean annual temperature (MAT), richness was increased in forest plots, and biomass was decreased in meadow plots; for mean annual precipitation (MAP) no effects were found. With higher plant species richness, insect biomass significantly decreased in forest and settlement plots and insect richness increased the latter one (Table 1). SV-SOS in forest plots did not mainly reflect plant species richness, since SV-SOS at the radius of 200 m was neither correlated to species richness of all vascular plants (Pearson correlation coefficient 0.01, *p* = 0.95) nor to richness of woody species only (Pearson correlation coefficient 0.17, *p* = 0.21).

**Table 1:** Effects of green-up variables, climate, land use, and plant species richness on insect BIN richness and Biomass.

	BIN richness	Biomass
	*gam: Negative Binomial, link = log*	*gam: gaussian, link = log*
	Estimate (Log-mean) * 10^3^ (St. error)	Estimate * 10^3^ (St. error)
Constant	73.00 (636.50)	−2433.1* (1128.2)
*Local land use (reference = Forest)*		
Meadow	1477.0* (598.2)	4815.4*** (935.8)
Arable field	988.7 (579.3)	3379.6*** (916.6)
Settlement	3323.4*** (730.8)	1483.0 (1384.4)
*Regional land use (reference = Semi-natural)*		
Agricultural	22.58 (28.25)	187.4*** (43.397)
Urban	23.01 (28.34)	−49.32 (46.646)
Mean SOS: Forest	10.01*** (2.672)	9.085* (3.900)
Mean SOS: Meadow	4.739* (2.327)	−8.314* (3.638)
Mean SOS: Arable field	5.261** (1.712)	−0.876 (2.447)
Mean SOS: Settlement	−2.691 (3.293)	−0.673 (6.724)
Spatial variability SOS: Forest	16.58*** (4.803)	37.66*** (7.118)
Spatial variability SOS: Meadow	−8.007* (3.130)	−19.11*** (5.016)
Spatial variability SOS: Arable field	−13.65*** (3.277)	−15.20** (5.068)
Spatial variability SOS: Settlement	−8.416** (3.233)	−13.86* (6.073)
Plant species richness: Forest	0.899 (0.737)	−3.887*** (1.003)
Plant species richness: Meadow	0.732 (0.810)	−2.449 (1.271)
Plant species richness: Arable field	1.279 (0.951)	0.808 (1.451)
Plant species richness: Settlement	2.811*** (0.720)	−2.755* (1.192)
MAP: Forest	0.157 (0.185)	0.174 (0.355)
MAP: Meadow	−0.053 (0.191)	0.052 (0.372)
MAP: Arable field	0.204 (0.252)	−0.007 (0.467)
MAP: Settlement	−0.026 (0.202)	0.635 (0.382)
MAT: Forest	150.4*** (42.759)	124.40 (77.553)
MAT: Meadow	30.63 (48.040)	−218.9** (80.832)
MAT: Arable field	50.90 (49.736)	−152.5 (85.402)
MAT: Settlement	9.169 (51.736)	68.19 (94.818)
Local temperature: Forest	37.77*** (11.153)	91.11*** (16.075)
Local temperature: Meadow	44.67*** (10.874)	88.52*** (15.839)
Local temperature: Arable field	36.10** (10.979)	92.21*** (16.430)
Local temperature: Settlement	28.40* (11.09)	72.71*** (18.078)
Local humidity: Forest	−1.123 (2.534)	−3.950 (4.039)
Local humidity: Meadow	4.000 (3.000)	5.237 (4.819)
Local humidity: Arable field	5.915* (2.834)	−0.011 (4.636)
Local humidity: Settlement	−8.553** (3.306)	0.912 (5.752)
Observations	1214	1293
Adjusted R^2^	0.381	0.581
Note:	Bold is significant: *p < 0.05; **p < 0.01; ***p < 0.001	

Estimates of the predictors of the generalized additive models to explain insect BIN Richness and Biomass over a whole season (8 samplings at 179 traps) in which regional land use and the interactions between local land use and MAP (long-term mean annual precipitation), MAT (long term mean annual temperature), local temperature, local humidity, mean SOS, spatial variability in SOS and plant species richness, were used as fixed linear effects, day as smoothed effect and space as random effect. Estimates for the variables in interaction with local land-use type represent the partial effects for the respective local land-use type, so they are independent of a reference. An offset of log(sampling days) was used to control for sampling period differences. For biomass, family = gaussian(link = “log”) and for richness, family = negative binomial was used. Standard errors (*10³) are indicated in between parentheses.

**Fig. 3:**
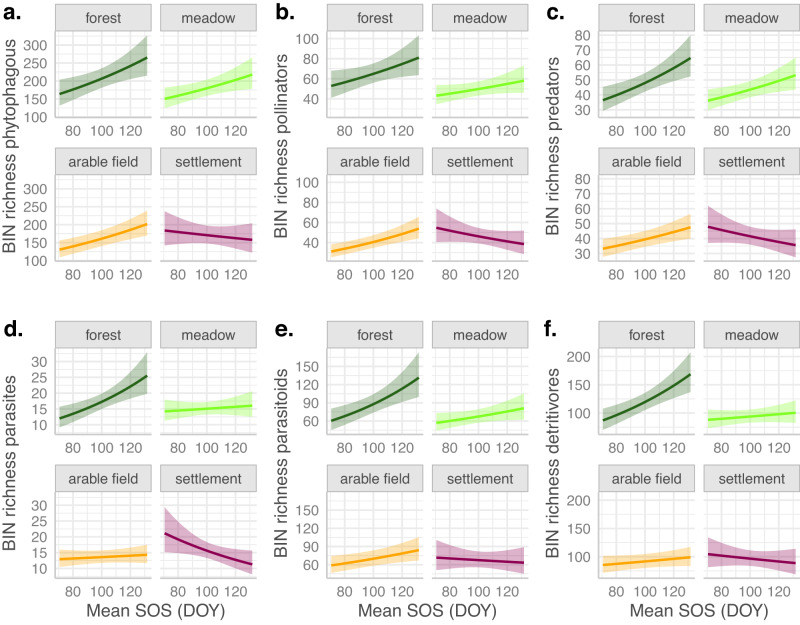
Partial effect plots of mean SOS in interaction with local land use on BIN richness per functional group. Effects were derived from generalized additive models (gam) in which we included regional land use and interactions of local land use with all green-up variables, with all-climate variables and with species richness as fixed linear effects. As in the initial model (Fig. 2, Table 1) ‘Day’ was used as smoothed effect and space as random effect. An offset of log(sampling days) was used to control for sampling period differences. Family = negative binomial was used. Insect data were recorded over a whole season (8 samplings at 179 traps), resulting in 1214 observations per model. **a**, phytophagous insects. **b**, pollinators. **c**, predators. **d**, parasites. **e**, parasitoids. **f**, detritivores.

**Fig. 4:**
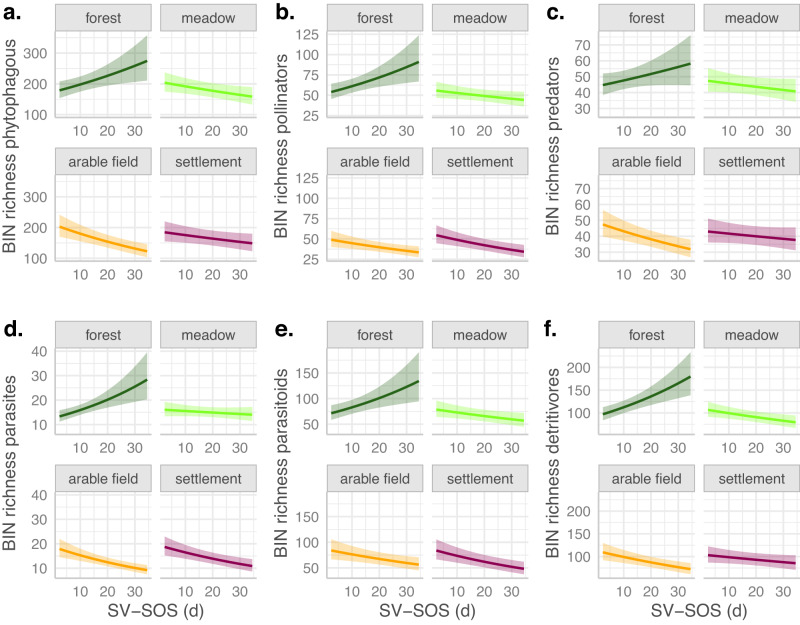
Partial effect plots of SV-SOS in interaction with local land use on BIN richness per functional group. Effects were derived from generalized additive models (gam) in which we included regional land use and interactions of local land use with all green-up variables, with all-climate variables and with species richness as fixed linear effects. As in the initial model (Fig. 2, Table 1), ‘Day’ was used as smoothed effect and space as random effect. An offset of log(sampling days) was used to control for sampling period differences. Family = negative binomial was used. Insect data were recorded over a whole season (8 samplings at 179 traps), resulting in 1214 observations per model. **a**, phytophagous insects. **b**, pollinators. **c**, predators. **d**, parasites. **e**, parasitoids. **f**, detritivores.

Generally, the partial effects of both green-up variables on BIN richness were quite similar among the different functional groups: phytophagous insects, pollinators, predators, parasites, parasitoids, and detritivores (Figs. 3, 4). In forests, the richness of almost all groups was significantly higher with a later mean SOS and a higher SV-SOS (*p* < 0.05), only for predators, the response to SV-SOS was not significant (*p* = 0.11). In arable fields, the richness significantly increased with mean SOS for phytophagous insects, pollinators, predators and parasitoids (*p* < 0.05). The increase was not significant for parasitoids and detritivores. In meadows, the richness increased significantly only for phytophagous insects and predators (*p* < 0.05), but insignificantly for pollinators, parasites, parasitoids and detritivores. In settlement sites, richness decreased with mean SOS, but only significantly for parasites (*p* < 0.05, Fig. 3). In contrast to forest plots, richness decreased with SV-SOS in meadows, arable field and settlement plots (Fig. 4). However, in meadows, this decrease was not significant for pollinators and parasites. In arable fields, this decrease was not significant for predators. In settlements, this decrease was not significant for phytophagous insects, predators and detritivores.

For most taxonomic groups, richness in forests was also significantly higher with a later mean SOS and with a higher SV-SOS (*p* < 0.05), but for groups with low amount of species this response was weak and insignificant (Orthoptera (*p* = 0.32), Rest (*p* = 0.06) and Red-Listed (*p* = 0.91) for mean SOS and Orthoptera (*p* = 0.08), Rest (*p* = 0.07) and Hemiptera (*p* = 0.67) for SV-SOS; Supplementary Figures 2, 3). In meadows, the only significant responses were higher richness with later mean SOS for Hemiptera, Rest and Red-Listed and lower richness with higher SV-SOS for Diptera and Red-Listed species (*p* < 0.05). In arable fields, richness was significantly higher with increased mean SOS with Coleoptera, Diptera, Hymenoptera, Lepidoptera, Rest and Red-Listed species and richness was decreased with increased SV-SOS for Diptera, Hymenoptera, Lepidoptera and Red-Listed species. In settlements, the response of richness to mean SOS was mostly insignificant, except for an increased richness with later mean SOS for Orthoptera. Richness decreased with SV-SOS for Hemiptera, Hymenoptera, Lepidoptera and Red-Listed species in settlements (Supplementary Figure 2).

**Fig. 5:**
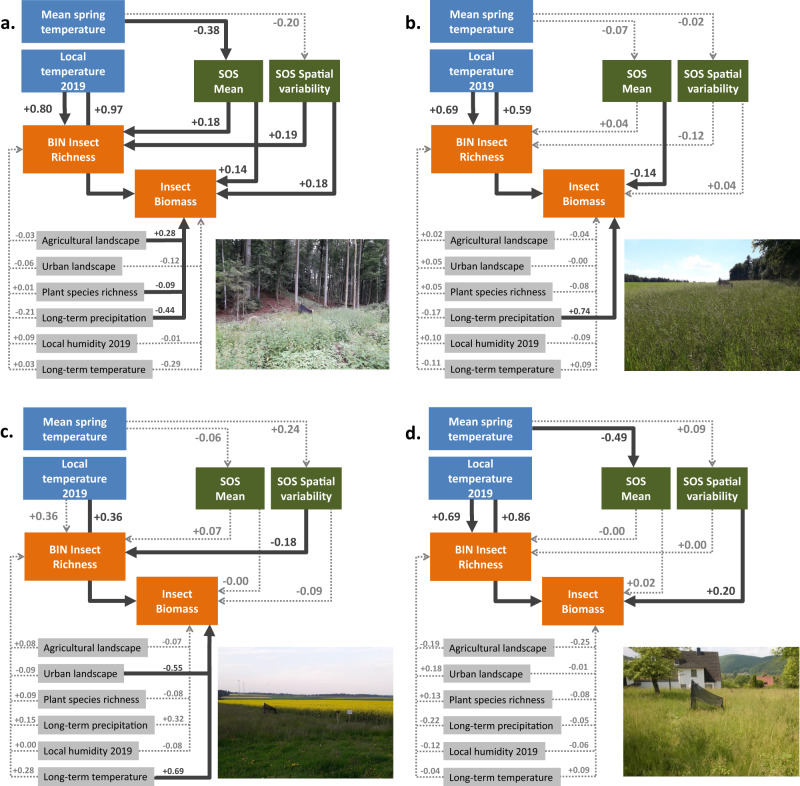
Confirmatory path models for each local land-use type. **a**, forest. **b**, meadow. **c**, arable field. **d**, settlement. Each confirmatory path model contains two components: one part using linear models to explain green-up variables by mean spring temperature. In forests, *n* = 55 per model, in meadows, *n* = 45 per model, in arable fields, *n* = 44 per model, in settlements, *n* = 35 per model; the other part using generalized additive models to explain BIN richness and biomass by the green-up variables, climate variables, regional land use and plant species richness as fixed linear effects. As in the initial model (Fig. 2, Table 1), ‘Day’ was used as smoothed effect and space as random effect. Instead of offsets of log(sampling duration), log(richness)/log(sampling duration) and log(biomass)/log(sampling duration) were used to control for sampling period differences. For biomass, family = gaussian(link = “log”) and for richness, family = negative binomial was used. In forest plots, *n* = 376 for richness and n = 395 for biomass, in meadows, *n* = 310 for richness and *n* = 333 for biomass, in arable fields, *n* = 298 for richness and *n* = 313 for biomass. In settlement plots, *n* = 230 for richness and *n* = 252 for biomass. Numbers indicate standardized partial effects. Dark grey thick arrows indicate significant paths (*p* < 0.05), light grey insignificant paths. Pictures show Malaise trap plots in each of the local land-use types.

To understand the diverging effects of temperature, directly versus indirectly via the green-up variables, we finally performed confirmatory path analyses (CPA, Fig. 5). With a separate model for each local land-use type, we tested the direct effects of spring temperature on the green-up variables in combination with the effects of the green-up variables to explain insect richness and biomass, together with climate variables, regional land use and plant species richness (see Methods). In forest plots, mean SOS was earlier with higher spring temperatures, and earlier mean SOS was associated with reduced insect richness and biomass. Our path model revealed a tendency (however *p* = 0.14) for a negative effect of higher spring temperatures on SV-SOS. Additional linear models which we ran for all forested areas within the 100 m radii and also for 1000 m radii around the 179 Malaise traps, confirmed this finding, as SV-SOS tended to decrease with higher spring temperatures for the 100 m radii (estimate ˗0.90, *p* = 0.06, adj. R2 = 0.02), and more strongly and significantly for the 1000 m radii (estimate ˗2.01, *p* < 0.001, adj. R2 = 0.22; Supplementary Figure 4). In turn, lower SV-SOS was associated with reduced insect richness and biomass. Thus, in forests, for both pathways of mean SOS and SV-SOS, spring temperature (indirectly) had a net negative effect on insect richness and biomass. This result markedly opposes the direct strong positive effects of local temperature as well as the insignificant effects of MAT (Fig. 5a). As in the original model, plant species richness had no significant effect on insect richness, but was associated with reduced biomass. For the other three local land-use types (meadow, arable field, settlement, Fig. 5 b-d), we neither found any significant path from spring temperature via green-up to insects, nor of plant species richness.

## Discussion

The most important and innovative finding in our study on insect richness and biomass in a temperate region is that higher air temperatures may have indirect negative effects on insects via the spring green-up pathway. Interestingly, these effects were most pronounced in forest plots, which have comparatively less intensive land use and should therefore be less affected by land-use-driven insect declines^3,6^.

In forest plots, later spring green-up (mean SOS) was associated with both higher insect richness and biomass (Table 1, Fig. 2), suggesting that – in line with our hypotheses – early leaf unfolding and flowering, which also occur under climate warming conditions, are unbeneficial. This could be explained either by the declining food quality over time and/or by phenology-related micrometeorological changes across the vertical habitat structure due to earlier canopy closure.

Regarding the first explanatory pathway, our results suggest that insect populations are less affected by a lack of forage before the SOS than by deterioration in its nutritional quality after SOS. Spring 2019 was relatively warm (+ 3.0  °C compared to 1961–1990 in March and +2.4 °C in April for Bavaria)^39^. If insect phenology responded more slowly to this warm spell than plant phenology, which is generally expected^17,40^, a mismatch in forage availability would be less likely than a quality impairment of the available food. Consequently, in opposite direction, the later the green-up takes place, the better it should be for most (herbivory) insects, as nutritional quality decreases over time^23^. Nevertheless, there are also examples for trophic interactions where the relevant phenology of primary consumers (e.g. egg hatching of winter moth^21^) shifts more than their resources (e.g. leaf emergence)^41^, but this trophic mismatch varies from year to year^21,42^. However, even in this example, the net population fitness was higher when green-up occurred later than egg hatching (although this led to larval starvation), while green-up before egg hatching had more detrimental effects due to lower fecundity and pupal weight^26^. In this respect, those results differ from our linear effect of mean SOS on the whole insect community, but *O. brumata* has a rather specific life cycle in which the larval stage coincides with budburst and leaf elongation. In contrast, we took representative snapshots of local flying insect communities in a ~90.000 km² region throughout the spring/summer season. This means that we also included phytophagous insects that have their larval stage later in the year (like in the study of Pöyry et al.^43^) and multivoltine species with at least one larval stage later in the year. We assume that for those species, nutrient quality is a more crucial factor than forage availability. Furthermore, our data contained species groups such as micro-Lepidoptera whose life history is unknown in detail^27^, and secondary consumers that are less dependent on vegetation^17,40^.

Secondly, a later onset of green-up in forests could influence insect richness and biomass through microclimatic conditions in the stands: When the tree canopy closes in spring after leaf unfolding and thus provides more shade to the stand interior, daytime temperatures in the understory are significantly lower than above the canopy or in open areas^44^. Consequently, from an insect’s perspective of which many do not rely on canopy greenness itself, but on geophytes and other understory vegetation, later canopy green-up, which allows longer warmer maximum temperatures inside deciduous stands and longer flowering of geophytes on the forest floor^45^ before green-up, should be beneficial for ectothermic and thermophilic insects^3,8^, as well as pollinators.

In forest plots, spatial variability in green-up (SV-SOS) was associated with higher insect biomass and richness (Table 1, Fig. 2), probably because higher variability provides high-quality vegetative resources over a longer period of time^14,29,30^. This spatial variability in green-up may result from species diversity in (woody) plants^46^, diversity of stand structure including shrubs and gaps^47^, and topographic heterogeneity, which has been shown to extend the flowering period of pollinator resources^28^, reducing temporal mismatch^33^ and improving pollinator diversity^28,48^. Since we included plant species richness itself in the models, which was positively but not significantly related to insect richness and negatively related to insect biomass in forest plots (Table 1), we were able to disentangle the species diversity effect from the spring green-up variability as such. To the best of our knowledge, this is the first time that this positive effect of green-up variability has been demonstrated for insects, which was previously only known for some large herbivores such as caribou^31^ and red deer^32^.

Considering the different functional groups, higher-order consumers are phenologically less sensitive than primary consumers (phytophagous insects and pollinators)^17–19^. Furthermore, a recent study showed that plant species richness has stronger effects on herbivorous than on predatory insects^49^. Our results in forest plots confirm this phenological response variation with functional groups: ‘Predators’ was the only functional group whose richness showed an insignificant response to SV-SOS in contrast to the significantly positive response of primary consumers (phytophagous insects and pollinators) to SV-SOS (Fig. 4). However, we found that the richness of detritivores, parasites and parasitoids, also higher trophic level groups, responded significantly positive to SV-SOS, similarly to the primary consumers (Fig. 4). One explanation for this could be that predators are usually generalists, while parasitoids, for example, are more often dependent on very specific hosts and therefore show similar responses to pollinators and phytophagous insects^34^. Regarding the effects of mean SOS on richness, however, we did not find differences among functional groups in forests. The fact that the richness of some taxonomic groups was not significantly responding to mean SOS and SV-SOS in forests can be attributed to the low species numbers in those groups (Supplementary Figures 2, 3).

In arable field, meadow and settlement plots, the effects of the green-up variables SOS and SV-SOS in a 100-m radius on insect richness and biomass were either less strong but in line with our hypotheses, insignificant, or even opposite to our hypotheses. There are many, albeit vague, suggestions to explain this (see Supplementary Discussion, Supplementary Figure 5).

Since we only used the spatial variation in temperature (and related variation in the SOS variables) across Bavaria in a space-for-time approach instead of a time series, our results are only to a limited extent transferable to climate change effects. However, they still suggest that climate change may further negatively impact (flying) insects via different pathways related to green-up. Mean SOS was earlier and SV-SOS was lower in forest plots with higher spring temperature (Fig. 5, Supplementary Figure 4), which is consistent with previous studies reporting that spring green-up is advancing with warming^12,13^ and that spatial variability in green-up decreases with warming at scales relevant to forage horizons of higher trophic levels^31,50–52^. Therefore, for both green-up pathways (mean SOS and SV-SOS), we expect that further climate change may have an indirect negative impact on insects, although the direct temperature influence would suggest a beneficial effect (Fig. 5).

According to our model results (Table 1, Fig. 2), insect declines could theoretically be alleviated by forests, which are greening-up later and whose green-up is more spatially diverse. Although later green-up could be achieved by increasing the proportion of woody species with lower temperature sensitivities^46,53^, this might probably alter insect community composition as such, particularly as herbivores are often specialized within plant orders or families. Higher spatial variability in spring green-up could be achieved by greater woody species diversity, the presence of shrubs or by more greater structural diversity with embedded openings, thus more diverse microclimates, as those may also be more diverse in insect resources^33,48^. This latter aspect could successfully contribute to a climate change adaptation strategy for insects in forests. One could assume that the modelled effects on insects as well as the adaptation strategies mentioned above are an indirect effect of (woody) plant species diversity rather than a direct effect of green-up variability. However, we can exclude this potential effect, since the green-up variables for forest plots were still significant even when plant species richness was included in the models. Moreover, neither plant species richness nor woody species richness was correlated with mean SOS and SV-SOS in 200-m radii (separate Pearson correlation tests).

So far, most studies which found trophic mismatch have focused on single species pairs, e.g. oak – winter moth^21^ and *Corydalis ambigua* – bumble bees^20^, even though the insect community as a whole has declined dramatically^1^. Furthermore, meta-analyses have examined phenological sensitivities across multiple species^17–19^, but not their ecological outcomes such as species richness and biomass. This is the first study to link vegetation phenology to overall richness and biomass of insects across an entire region, thereby filling an important research gap. Until recently, the field of observational ecology was relatively disconnected from the field of remote sensing, despite its enormous potential due to its ability to cover large areas and its low labor intensity^36^ (but see the study of Pöyry et al.^43^). Especially the freely available Sentinel-2 data with its fine spatial and temporal resolution has the potential to change this. However, up to now, there has only been one study^54^ that used Sentinel-2-derived phenology parameters, including SOS to explain variability in insect populations. However, the study did not aim to link vegetation phenology per se to insects, but used phenology parameters as proxies for land-use intensity.

Our study has shown that spring green-up and its inherent spatial variability influence the insect community in temperate Europe, and that climate warming is likely to contribute to part of today’s dramatic insect decline through this pathway, especially in forests, the least intensive land-use type in our study. There, climate warming may contribute to a decline in insect richness and biomass through an earlier start and reduced spatial variability of spring green-up.

## Methods

### Study design

For our study, we used the study design developed by Redlich et al.^38^., which was also used by Uhler et al.^3^ to study land use and climate affects in a space-for-time approach. The 179 study plots were stratified across a climate and land-use gradient in Bavaria, southern Germany. Mean annual temperatures (MAT) at these sites ranged from 5.0 to 10.3 °C and mean annual precipitation (MAP) from 550 to 1961 mm (1991 – 2020). 60 quadrants of ~6×6 km, in which the plots were later established, had been selected according to a nested land-use intensity gradient: regarding regional land-use, 20 were in semi-natural landscapes (dominated by forest), 20 in agricultural landscapes (dominated by arable fields and grasslands) and 20 in urban landscapes (dominated by settlement area), based on CORINE land use (2018) from the Copernicus Land Monitoring Service. In each quadrant, three (in one quadrant only two because of permission constraints) plots were established in the three most dominant local land-use types. This resulted in 55 plots dominated by forest (forest plots), 45 plots dominated by grassland (meadow plots), 44 plots dominated by cropland (arable field plots) and 35 plots dominated by urbanized areas (settlement plots). For a spatial visualization of the study design, see Supplementary Figure 6. For a more detailed description, see Redlich et al.^38^.

### Insect data

An extended version of the insect dataset^55^ from Uhler et al.^3^ was used, which was obtained as follows. In each plot a black Townes-style Malaise trap (height front: 0.90 m; height rear: 0.60 m; length: 1.60 m)^56^ was placed to catch flying insects (in fact “arthropods”, since some minor species groups such as spiders were caught as well and were not excluded from the analysis). The sampling vessels were exchanged every two weeks between mid-April and mid-August 2019, resulting in eight catches per plot and a total of 1293 samples. Insect biomass was measured per catch and - as a proxy for species richness - BIN richness was determined by DNA barcoding, according to Uhler et al.^3^ and Hausmann et al.^57^. Since BIN clusters match taxonomic species well (90-99%)^3,57^, taxonomic and functional groups were easily defined based on BIN richness data. We distinguished the six largest taxonomic orders, specifically Coleoptera, Diptera, Hemiptera, Hymenoptera, Lepidoptera and Orthoptera, and six functional groups, namely phytophagous insects, pollinators, predators, parasites, parasitoids and detritivores. Species, which are phytophagous as larvae but pollinator as adults (Lepidoptera) were assigned to both groups. Furthermore, we separated Red-Listed species, based on Red Lists for the federal state of Bavaria and Germany. For a more detailed description of the plot establishment, field and lab work, see Uhler et al. (2021)^3^.

### Spring green-up data

As a measure of spring green-up, we used start-of-season (SOS) data from the High-Resolution Vegetation Phenology and Productivity (HRVPP) product offered by the Copernicus Land Monitoring Service^37^ which is based on remotely sensed data from Sentinel-2 with a 10 m spatial resolution and a 3 to 5-day temporal resolution. For further details, see Zhanzhang et al.^37^. On Plant Phenology Index (PPI) time series (see also Jin & Eklundh^58^), a winter gap-filling algorithm and a double-logistic curve-fitting is applied. Based on the resulting curves, regular 10-day seasonal trajectories are derived. Then a ‘season identification’ identifies per pixel if either one or two seasonal curves are represented. Accordingly, SOS is defined as the Day of the Year (DOY) at which the curve exceeds 25% of the amplitude of the first season^59^.

We initially derived SOS data (2017–2019) for 100, 200, 500, 1000, 2000 and 3000 m around the 179 plots, using the raster package^60^ and the sp package^61^ in R version 4.1.2^62^, but we only kept the smallest radius of 100 m in the analysis which had throughout the strongest effects in the models. After filtering out the pixels, which were overlapping with water, we derived two different green-up metrics per plot: the mean SOS of all pixels (2017–2019) and the spatial variability of SOS as the standard deviation of SOS for all pixels per year, averaged over 2017–2019 (SV-SOS). For a better understanding, Supplementary Figure 1 demonstrates selected examples of SOS distributions over space and a single year (2019) for two different forest and arable field plots. The reason why we used not only 2019 but also the two preceding years is that the insect population at a given location and year is never just a consequence of circumstances (in this case the phenology) at that time, but is rather affected by the long-term habitat conditions (including the phenology of other years).

### Climate data

To explain green-up data by spring temperatures, we calculated the mean temperature of March and April during 2017-2019 from gridded monthly averaged mean daily air temperature (°C) on a spatial resolution of 1 km from the German Meteorological Service (Deutscher Wetterdienst, DWD)^63^ and assigned each plot to the nearest 1 km x 1 km grid cell. Furthermore, long-term MAT and MAP, which were directly derived from an already published dataset^64^, were calculated in the same way^38^. Hourly measurements of local air temperature and local air humidity in 2019 were derived using ibutton thermologgers (type DS1923) at the plot, mounted at 1.10 m height, facing north, under a roof panel to avoid direct sun exposure. As Uhler et al.^3^ did, these four climate variables were used for modelling insect biomass and richness (see statistical analysis). For modelling, hourly measurements were averaged per sampling period in 2019.

### Land use around the plots

In order to come to our study design^38^, to get a better understanding of SOS distributions among land-use classes (Fig. 1), and to test more in depth climate effects on green-up variables for forested area in all 179 plots (Supplementary Figure 4), a corresponding shapefile of land use was created^64^. The shapefile combines ATKIS data (2019) from the Landesamt für Digitalisierung, Breitband und Vermessung and CORINE (2018) from the Copernicus Land Monitoring Service. 99.9% of all studied non-water pixels in 100-m radii around the plots were covered by four main land-use classes. More specifically, 35.3% were covered by forests, 21.0% by grassland, 25.6% by cropland, and 17.0% by urbanized areas. Besides, we used InVeKoS data from the Bayerische Landesanstalt für Landwirtschaft (LfL) to investigate the SOS distribution of maize specifically.

### Plant richness data

We assumed plant species richness to correlate with spatial variability in SOS, and therefore we wanted to distinguish the species richness effect from the spring green-up effect per se in our models. To do so, we made use of the vascular plant species richness dataset^65^ from Tobisch et al.^66^, which was obtained by vegetation surveys in a 200-m radius around the Malaise trap plots. Between mid-May and the end of July 2019, vegetation was sampled in seven 10 m² subplots directly surrounding the Malaise traps. Additionally, species pools within a 200-m radius were assessed between mid-May and early August 2020 using standardized transect walks in which walking time was proportional to the area percentages of dominant habitat types within the 200-m radius, and walking time was 60 minutes for each plot. Total plant species richness was obtained from all unique species encountered in at least one of the two sampling rounds^66^. This variable was additionally used as predictor in our insect models.

### Statistics and reproducibility

To test the partial effects of mean SOS an SV-SOS (2017-2019, see spring green-up data) at the plots in explaining insect biomass and richness, we took the same generalized additive models used by Uhler et al.^3^ with regional land use, local land use, MAT, MAP, local temperature and local humidity as predictors using the mgcv package^67^ in R version 4.1.2^62^. Then, we added mean SOS and SV-SOS, their interaction with local land use (i.e. forest, arable field, meadow, and settlement) because we expected differences among them, as well as plant species richness. Furthermore, we expected that other (climatic) variables could indirectly interact with green-up to explain insect richness and biomass (e.g. higher temperatures lead to earlier green-up). Therefore, we included also for MAT, MAP, local temperature, local humidity and plant richness, their interaction with local land-use type. As Uhler et al.^3^ did, we added the mean day of a sampling period as a smooth non-linear predictor to account for seasonality, an offset of sampling duration to control for variation among sampling periods, and a smooth plot-specific intercept based on its location, to correct for (non-linear) spatial correlation.

To test whether or not SV-SOS was actually representing (woody) plant species richness, which was only recorded at 200-m radii, we also did a Pearson correlation test between SV-SOS at the 200-m radius and plant species richness per land-use type. Additionally, we did the same test with species richness of only the woody species, as these are expected to be captured mostly by Sentinel-2. Subsequently, we ran this model type for each single functional and taxonomic group individually, to explore if green-up effects on insect richness differed among them.

To show how indirect temperature effects by the green-up pathway differed from their direct effects on insect richness and biomass, we used confirmatory path models (CPM), i.e. structural equation models (SEM)^68^. For this analysis, we did not apply interactions with local land-use type, but instead we ran separate models for each local land-use type. To explain mean SOS and SV-SOS, we used spring temperature in linear models. To explain insect biomass and richness subsequently, we used mean SOS and SV-SOS, MAT, MAP, local temperature, local humidity, plant species richness and regional land-use type in generalized additive models.

However, as it is not possible to directly combine generalized additive models and linear models in one CPM, we standardized estimates of these individual models by centering them to their mean and scaling them to their standard deviation using the function standardize_parameters within the parameters package^69^ in R^62^. We visually connected the different estimates afterwards. For the generalized additive model for biomass, biomass was replaced by log(Biomass)/log(sampling duration) and in the insect BIN richness model, BIN richness was replaced by log(BIN richness)/log(sampling duration), because the usage of the offset(duration) was not allowed.

Although we only presented the path model results for the 100-m radii, because the green-up variables explained insect variability best at this scale, we modeled green-up variables by spring temperature per local land-use type, using simple linear models, also for higher radii, and presented the output for 1000-m radii (Supplementary Figure 4). Additionally, because in all insect models green-up effects on insects were strongest in forest plots, and we aimed at the full understanding of processes occurring in forests, we ran simple linear models to explain the two green-up variables in all forested areas (see ‘Land use around the plots’) by spring temperature.

### Reporting summary

Further information on research design is available in the Nature Portfolio Reporting Summary linked to this article.

### Supplementary information

Supplementary Information

Reporting Summary

## Data Availability

All raw data for the analyses of this study are publicly available on figshare under 10.6084/m9.figshare.24228892.

## Code availability

Annotated R code to reproduce the statistical analyses and figures of this study are publicly available on figshare under 10.6084/m9.figshare.24228892.
