# Supplementary material for: Earlier and more uniform spring green-up linked to lower insect richness and biomass in temperate forests
Source: Commun Biol. 2023 Nov 7;6:1052. doi: 10.1038/s42003-023-05422-9 (PMC10630471; doi:10.1038/s42003-023-05422-9)
Supplement: Supplementary file 1 — Supplementary Information [file 42003_2023_5422_MOESM1_ESM.pdf]

Supplementary information for

Earlier and more uniform spring green-up linked to lower insect richness and biomass in temperate forests

Lars Uphus, Johannes Uhler, Cynthia Tobisch, Sandra Rojas-Botero, Marvin Lüpke, Caryl Benjamin, Jana Englmeier, Ute Fricke, Cristina Ganuza, Maria Haensel, Sarah Redlich, Jie Zhang, Jörg Müller, Annette Menzel

Supplementary information:

Supplementary Discussion
Supplementary References
Supplementary Figures

Supplementary Discussion

In arable field, meadow and settlement plots, the effects of all green-up variables in a 100-m radius on insect richness and biomass were either less strong but in line with our hypotheses, insignificant, or even opposite to our hypotheses. There are many, albeit vague, suggestions to explain this. Green-up should have a lower impact if the quantity or quality of primary resources is of lower importance for (flying) insects in Malaise traps, or if certain methodological constraints apply to these land-use types. In settlements plots, both biomass and richness did generally not respond to mean SOS (except the richness of parasites which increased with advanced SOS), probably because "green" is often cultivated (e.g. lawns with only a few species) and pixels are more often a mixture of green and sealed area [1].

To mean SOS, we saw diverging responses of richness and biomass in arable fields and meadows. In contrast to forests, biomass in meadows was increased with an earlier mean SOS, probably because these meadows were also highly productive in nutritional resources to insects [2]: where plant biomass is higher, insect biomass is also higher. This effect was not significant in arable fields, probably trough the higher intensity of pesticides [3]. In contrast to the effects on biomass, we found a significant effect of later mean SOS increasing insect richness in meadows and arable field plots (Table 1, Fig. 2), which is in line with [4] who found a positive correlation between a later SOS of winter wheat and bumblebee abundance. The authors argue that the main mechanism behind this is that an earlier SOS reflects faster growth of winter wheat (the only crop regarded in the study), which should be related to increased nitrogen input, i.e. higher land-use intensity, leading to lower plant species richness and thus lower bumblebee richness. This pathway could be an explanation for our result in arable fields and meadows as well. In arable fields, this effect was found for most functional groups, but not significantly for parasites and detritivores. In meadows, this effect was not significant for other groups than phytophagous insects and predators. In forest plots, however, with more extensive land use, this positive effect of mean SOS was even stronger and could be interpreted by e.g. the phenology itself and the concept of trophic mismatch (See Main).

With a higher SV-SOS, biomass and richness were significantly decreased in meadows (however not for pollinators and parasites), arable fields (however not for predators) and settlements (however not for phytophagous insects, predators and detritivores), in contrast to forests. Since the within-field spatial variability in SOS is low, most of the SV-SOS within arable field and meadow plots can be attributed to different crops and possibly other land-use classes. In this sense, crop types with a very pronounced early or late SOS when co-occurring with other crops would likely increase SV-SOS and then

have a positive impact on biodiversity [5, 6], but this was not the case in our study on insects. Here, a closer look at the crop data showed that maize in particular, which is known to be less suitable for insects than other crops [7], contributed to both late SOS (mean DOY of 148.6 in 2017-2020) and high variability in SOS (Supplementary Figure 5).

Supplementary References

1. Uhler J*, et al.* Relationship of insect biomass and richness with land use along a climate gradient. *Nature Communications* **12**, 5946 (2021).

2. Haddad NM, Tilman D, Haarstad J, Ritchie M, Knops JM. Contrasting effects of plant richness and composition on insect communities: a field experiment. *The American Naturalist* **158**, 17-35 (2001).

3. Sánchez-Bayo F, Wyckhuys KA. Worldwide decline of the entomofauna: A review of its drivers. *Biological Conservation* **232**, 8-27 (2019).

4. Abdi AM*, et al.* Biodiversity decline with increasing crop productivity in agricultural fields revealed by satellite remote sensing. *Ecological Indicators* **130**, 108098 (2021).

5. Fahrig L*, et al.* Functional landscape heterogeneity and animal biodiversity in agricultural landscapes. *Ecology letters* **14**, 101-112 (2011).

6. Sirami C*, et al.* Increasing crop heterogeneity enhances multitrophic diversity across agricultural regions. *Proceedings of the National Academy of Sciences* **116**, 16442-16447 (2019).

7. Hass AL, Brachmann L, Batáry P, Clough Y, Behling H, Tscharntke T. Maize-dominated landscapes reduce bumblebee colony growth through pollen diversity loss. *Journal of Applied Ecology* **56**, 294-304 (2019).

Supplementary Figures

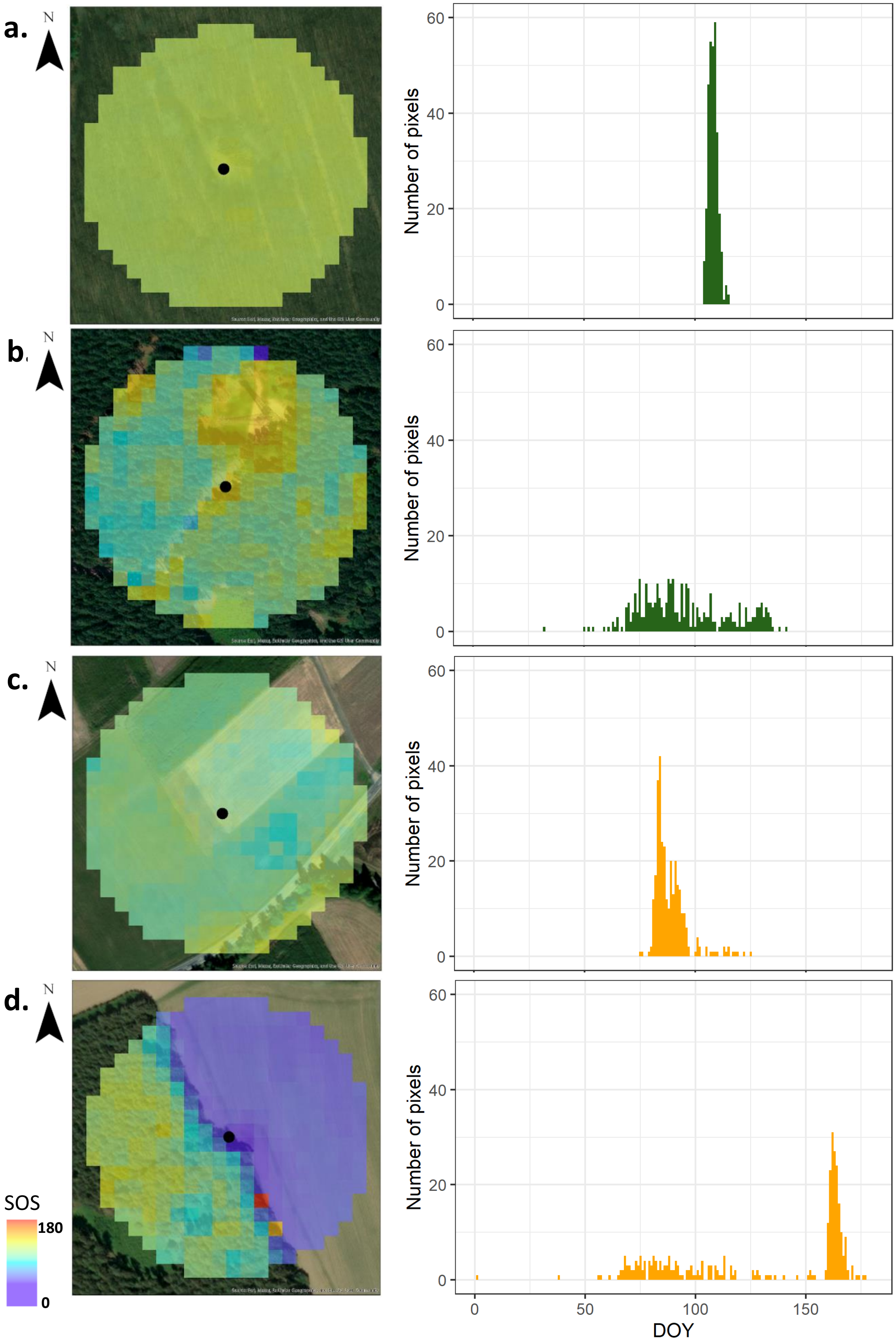

Supplementary Figure 1. The distribution of SOS in some example plots in the year 2019. **a**, a forest plot with low spatial variability. **b**, a forest plot with high spatial variability. **c**, an arable field plot with low spatial variability. **d**, an arable field plot with high spatial variability. In each panel, the left hand feature shows the SOS for each 10 m pixel in a 100 m radius around the malaise trap. The right hand feature shows the temporal distribution of SOS.

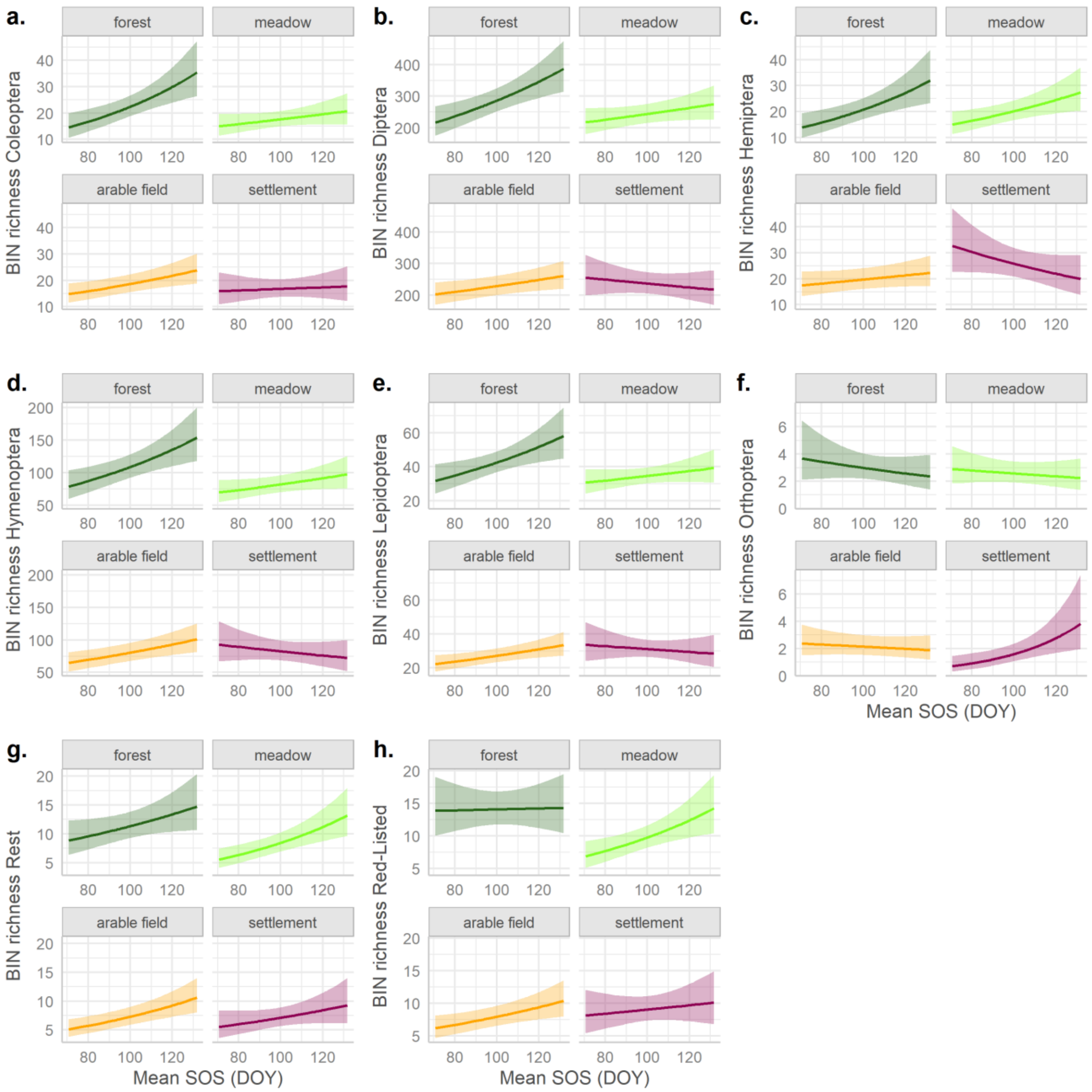

Supplementary Figure 2. **Partial effect plots of mean SOS in interaction with local land use on BIN richness per taxonomic group.** Effects were derived from generalized additive models (gam) in which we included regional land use and interactions of local land use with all green-up variables, with all climate variables and with species richness as fixed linear effects. As in the initial model (Fig.2, Table 1), 'day' was used as smoothed effect and space as random effect. An offset of log(sampling days) was used to control for sampling period differences. Family = negative binomial was used. **a**, Coleoptera. **b**, Diptera. **c**, Hemiptera. **d**, Hymenoptera. **e**, Lepidoptera. **f**, Orthoptera, **g**, species belonging to other taxa. **h**, Red-Listed species across taxa.

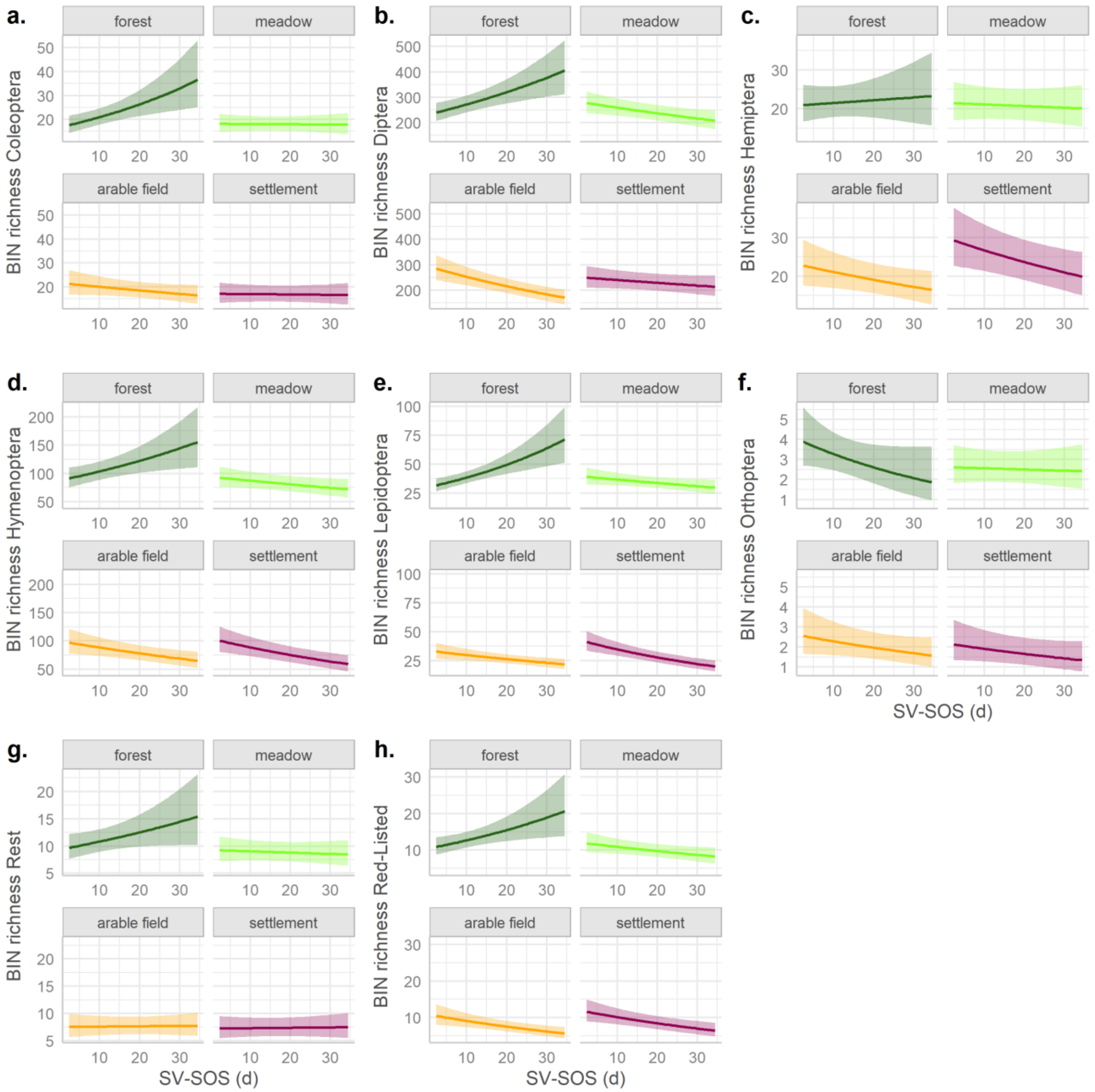

Supplementary Figure 3. **Partial effect plots of SV-SOS in interaction with local land use on BIN richness per taxonomic group.** Effects were derived from generalized additive models (gam) in which we included regional land use and interactions of local land use with all green-up variables, with all climate variables and with species richness as fixed linear effects. As in the initial model (Fig.2, Table 1), 'day' was used as smoothed effect and space as random effect. An offset of log(sampling days) was used to control for sampling period differences. Family = negative binomial was used. Insect data were recorded over a whole season (8 samplings at 179 traps), resulting in 1214 observations per model. **a**, Coleoptera. **b**, Diptera. **c**, Hemiptera. **d**, Hymenoptera. **e**, Lepidoptera. **f**, Orthoptera, **g**, species belonging to other taxa. **h**, Red-Listed species across taxa.

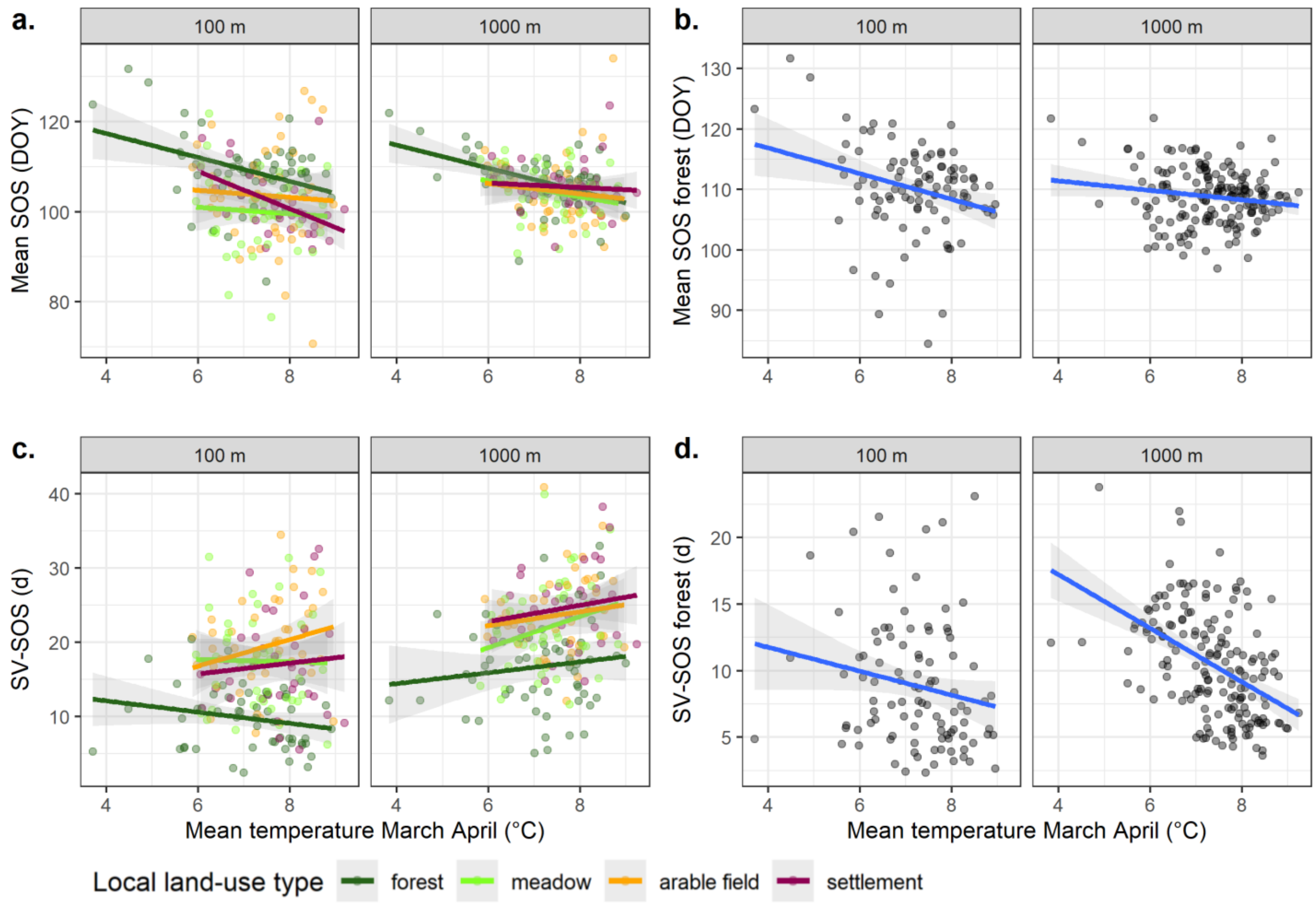

Supplementary Figure 4. **Effects of spring temperature on green-up variables.** Linear effects of mean March and April temperature (2017-2019) (°C) on the spring green-up variables for 100-m and 1000-m radii around the Malaise trap plots. **a**, effects on mean SOS of the entire plot radii. **b**, effects on mean SOS of only forest area within the plot radii. **c**, effects on SV-SOS of the entire plot radii. **d**, effects on SV-SOS of only forest area within the plot radii. Colors (in **a**,**c**,**e**) represent local land-use type of the plots. N = 179 per radius per panel.

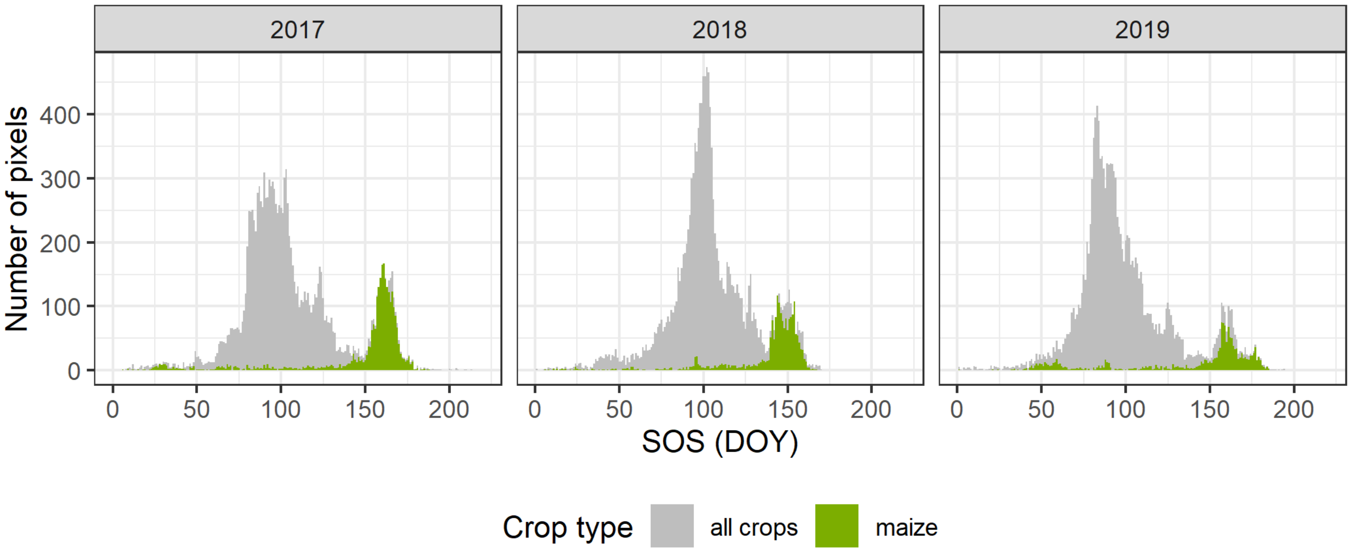

Supplementary Figure 5. **SOS of maize compared to other crops.** The annual distribution of SOS for pixels covering maize fields (green) on top of all cropland together (grey) per year (2017-2019) in 100m radii around the 179 Malaise trap plots.

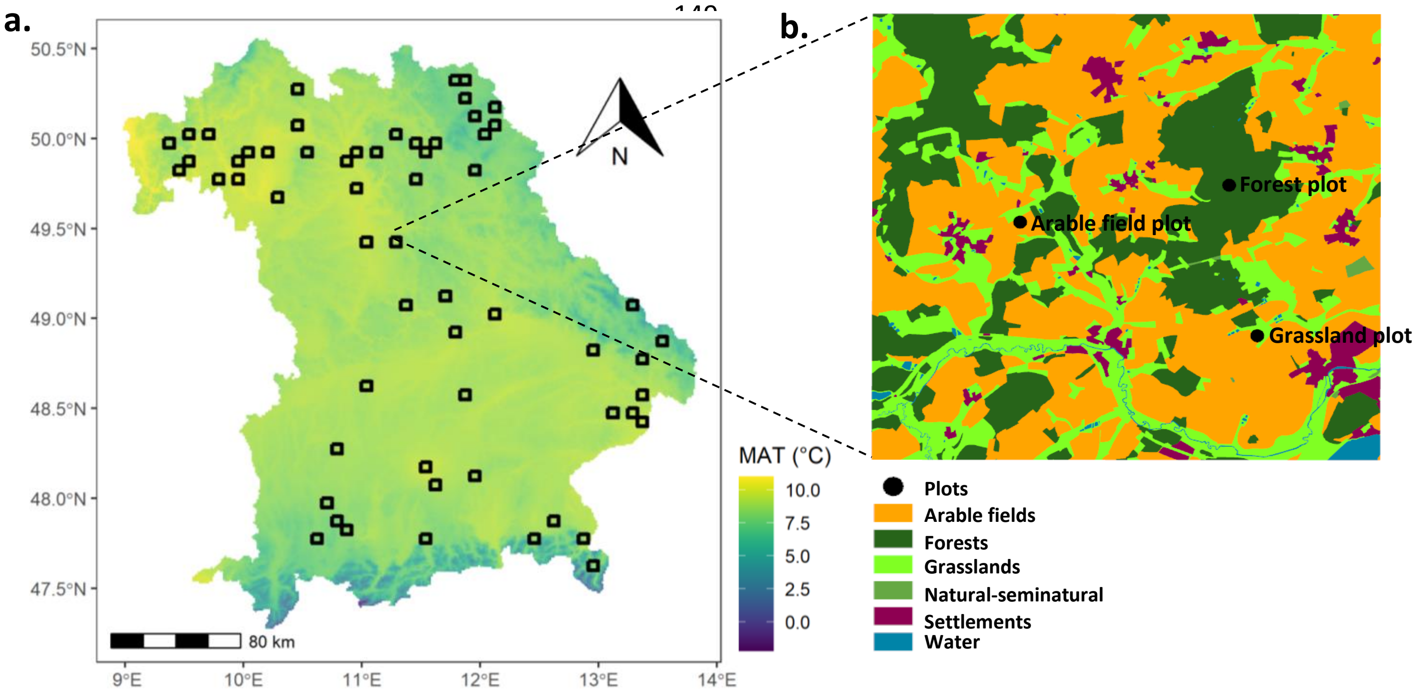

Supplementary Figure 6. **The nested study design**. **a**, the spatial distribution of 60 quadrants (black squares) throughout the federal state of Bavaria, Germany, on a regional land-use and temperature gradient. Background color represents mean annual temperature (MAT) over 1991-2020. **b**, the distribution of three study plots (black dots), based on local land use (here forest, arable filed and grassland) within an example quadrant with agriculture as regional land-use type.
