# Supplementary material for: Earlier and more uniform spring green-up linked to lower insect richness and biomass in temperate forests
Source: Commun Biol. 2023 Nov 7;6:1052. doi: 10.1038/s42003-023-05422-9 (PMC10630471; doi:10.1038/s42003-023-05422-9)
Supplement: Supplementary file 2 — Reporting Summary [file 42003_2023_5422_MOESM2_ESM.pdf]

nature portfolio

Corresponding author(s): Lars Uphus

Last updated by author(s): Oct 2, 2023

Reporting Summary

Nature Portfolio wishes to improve the reproducibility of the work that we publish. This form provides structure for consistency and transparency in reporting. For further information on Nature Portfolio policies, see our Editorial Policies and the Editorial Policy Checklist.

Statistics

For all statistical analyses, confirm that the following items are present in the figure legend, table legend, main text, or Methods section.

n/a	Confirmed	
☐	☒	The exact sample size (*n*) for each experimental group/condition, given as a discrete number and unit of measurement
☐	☒	A statement on whether measurements were taken from distinct samples or whether the same sample was measured repeatedly
☐	☒	The statistical test(s) used AND whether they are one- or two-sided<br>*Only common tests should be described solely by name; describe more complex techniques in the Methods section.*
☐	☒	A description of all covariates tested
☐	☒	A description of any assumptions or corrections, such as tests of normality and adjustment for multiple comparisons
☐	☒	A full description of the statistical parameters including central tendency (e.g. means) or other basic estimates (e.g. regression coefficient) AND variation (e.g. standard deviation) or associated estimates of uncertainty (e.g. confidence intervals)
☐	☒	For null hypothesis testing, the test statistic (e.g. F, t, r) with confidence intervals, effect sizes, degrees of freedom and P value noted<br>*Give P values as exact values whenever suitable.*
☒	☐	For Bayesian analysis, information on the choice of priors and Markov chain Monte Carlo settings
☐	☒	For hierarchical and complex designs, identification of the appropriate level for tests and full reporting of outcomes
☐	☒	Estimates of effect sizes (e.g. Cohen's *d*, Pearson's *r*), indicating how they were calculated

Our web collection on statistics for biologists contains articles on many of the points above.

Software and code

Policy information about availability of computer code

Data collection	No software was used for data collection
Data analysis	All data was done in R Version 4.1.2. All packages used are mentioned in the manuscript. Full R Code is provided here: https://figshare.com/s/1619487977acee069eb1 , which is mentioned in the manuscript

For manuscripts utilizing custom algorithms or software that are central to the research but not yet described in published literature, software must be made available to editors and reviewers. We strongly encourage code deposition in a community repository (e.g. GitHub). See the Nature Portfolio guidelines for submitting code & software for further information.

Data

Policy information about availability of data

All manuscripts must include a data availability statement. This statement should provide the following information, where applicable:

- Accession codes, unique identifiers, or web links for publicly available datasets
- A description of any restrictions on data availability
- For clinical datasets or third party data, please ensure that the statement adheres to our policy

All data supporting the findings of this study will be made publicly available on figshare upon publication. Until publication, data is accessible by the following link: https://figshare.com/s/1619487977acee069eb1

Human research participants

Policy information about studies involving human research participants and Sex and Gender in Research.

Reporting on sex and gender	NA
Population characteristics	NA
Recruitment	NA
Ethics oversight	NA

Note that full information on the approval of the study protocol must also be provided in the manuscript.

Field-specific reporting

Please select the one below that is the best fit for your research. If you are not sure, read the appropriate sections before making your selection.

☐ Life sciences ☐ Behavioural & social sciences ☒ Ecological, evolutionary & environmental sciences

For a reference copy of the document with all sections, see nature.com/documents/nr-reporting-summary-flat.pdf

Ecological, evolutionary & environmental sciences study design

All studies must disclose on these points even when the disclosure is negative.

Study description

We studied the effects of green-up variables (Sentinel-2 derived mean SOS and its spatial variability) on insect biomass and richness collected by Malaisetraps and identified by Metabarcoding along a land use and climate gradient.
We used the study design and extended version of the insect dataset of Uhler et al. (https://doi.org/10.1038/s41467-021-26181-3), in which 179 Malaise traps were set up in 2019 along a gradient of increasing land use intensity, ranging from forests, to meadows, to arable fields, and settlements. Malaise traps are versatile passive net-traps, capable of catching a large spectrum of insect taxa and is very effective when the aim is to get a representative snapshot of the local insect community. The analyzed sites were spatially distributed over 90.000km2 and covered an elevational gradient of 1000m. Generalized additive models were fitted to test for the effects of mean SOS and SV-SOS on insect biomass and richness, while adjusting for local and landscape-level land-use categories, local short-term and long-term climate variables, and plant species richness.

Research sample

A green-up sample in this study is a spatial raster of 10*10 cm pixels of SOS in a buffer zone of 100m per year (2017-2019) around each Malaise trap, resulting in 179 samples, from which mean SOS and SV-SOS were extracted per year and averaged over the three years.

A plant sample is the number of encountered vascular plants within 200 meters around each plot, resulting in 179 samples.

An insect sample in this study is the total amount of insects caught by a Malaisetrap at a given site over the course of two weeks and is considered representative for the insect community at the respective study site. Overall, we collected 1,293 insect samples (biomass) from which we obtained the meta-barcoding results of 1214 samples.

Sampling strategy

As Uhler et al. (https://doi.org/10.1038/s41467-021-26181-3) did, we collected data based on a stratified selected set of different habitats and climate conditions based on GIS analyses reported in Redlich et al (doi.org/10.1101/2021.03.05.434036).

Data collection

Green-up: Green-up was obtained by start of season (SOS) data from the High Resolution Vegetation Phenology and Productivity (HRVPP) product offered by the Copernicus Land Monitoring Service which is based on remotely sensed data from Sentinel-2 with a 10 m spatial resolution and a 3 to 5-day temporal resolution.

Plants: Plant species richness was obtained by vegetation surveys in a 200-m radius around the Malaise trap plots and was additionally used as predictor in our insect models. Between mid-May and the end of July 2019, vegetation was sampled in seven 10 m² subplots directly surrounding the Malaise traps. Additionally, species pools within a 200-m radius were assessed between mid-May and early August 2020 using standardized transect walks in which walking time was proportional to the area percentages of dominant habitat types within the 200-m radius, and walking time was 60 minutes for each plot. Total plant species richness was obtained from all unique species encountered in at least one of the two sampling rounds.

Insects: Insect samples were obtained using Malaise traps in the field weighed in the lab and subsequently meta-barcoded.

Timing and spatial scale

Insects: Sampling took place from May to August 2019 on 179 plots all across Bavaria, Germany.
Green-up: Sampling took place by Sentinel-2 in the years of 2017-2019
Plants: Sampling took place between mid-May and the end of July 2019. And afterwards between mid-May and early August 2020

Data exclusions	No data was exluded from the analysis, when data was available. In case data was not available, see "Disturbance"
Reproducibility	All data used for analysis is publicly available and allows full reproducibility of all results and graphs.
Randomization	The sampling followed a strict a prior selection of sites only based on GIS information and with sufficient replicates Redlich et al (doi.org/10.1101/2021.03.05.434036)
Blinding	Blinding is not possible in setting up insect traps (doi.org/10.1101/2021.03.05.434036), nor for vegetation sampling, transect walks, or deriving satellite data.

Did the study involve field work? ☒ Yes ☐ No

Field work, collection and transport

Field conditions	Insects: Malaise traps are passive traps and operated in all weather conditions from April to August. We measured local temperature and moisture with data loggers. These data are fully presented in our data and included in analyses<br>Plants were collected during the summer season, when all present plants were observable.
Location	The sampling plots are spread over the federal state Bavaria in South Eastern Germany. For further information regarding the study sites (e.g. coordinates) see the available data file.
Access & import/export	The material was collected with the permission of the nature conservation authorities of the governments Upper Franconia (17.04.2019), Lower Franconia (09.05.2019), Middle Franconia (19.03.2019), Lower Bavaria (10.05.2019), Upper Bavaria (15.05.2019), Swabia (12.03.2019), Upper Palatinate (02.05.2019).
Disturbance	For some sampling periods, several traps collapsed, or were destroyed. For other sampling periods, climate data was missing. See Supplementary Table 3 in Uhler et al. (https://doi.org/10.1038/s41467-021-26181-3) for further information.

Reporting for specific materials, systems and methods

We require information from authors about some types of materials, experimental systems and methods used in many studies. Here, indicate whether each material, system or method listed is relevant to your study. If you are not sure if a list item applies to your research, read the appropriate section before selecting a response.

Materials & experimental systems

n/a	Involved in the study	
☒	☐	Antibodies
☒	☐	Eukaryotic cell lines
☒	☐	Palaeontology and archaeology
☐	☒	Animals and other organisms
☒	☐	Clinical data
☒	☐	Dual use research of concern

Methods

n/a	Involved in the study	
☒	☐	ChIP-seq
☒	☐	Flow cytometry
☒	☐	MRI-based neuroimaging

Animals and other research organisms

Policy information about studies involving animals; ARRIVE guidelines recommended for reporting animal research, and Sex and Gender in Research

Laboratory animals	This study did not involve laboratory animals.
Wild animals	Arthropods were captured with Malaise traps in the field using ethanol as trapping and killing medium. The identification and further analysis required the killing of the arthropods.
Reporting on sex	Sex was not considered in this study.
Field-collected samples	The sampled arthropods were preserved in 80% ethanol.
Ethics oversight	The material was collected with the permission and ethical approval of the nature conservation authorities of the governments Upper Franconia, Lower Franconia, Middle Franconia, Lower Bavaria, Upper Bavaria, Swabia, Upper Palatinate.

We copied this statement from Uhler et al. (https://doi.org/10.1038/s41467-021-26181-3), from whom we obtained our insect dataset.

Note that full information on the approval of the study protocol must also be provided in the manuscript.
